# GBS-Based SNP Map Pinpoints the QTL Associated With Sorghum Downy Mildew Resistance in Maize (*Zea mays* L.)

**DOI:** 10.3389/fgene.2022.890133

**Published:** 2022-07-20

**Authors:** Kashmiri Prakash Jadhav, Gajanan R. Saykhedkar, Pandiampalayam Marappan Tamilarasi, Subramani Devasree, Rajagopalan Veera Ranjani, Chandran Sarankumar, Pukalenthy Bharathi, Adhimoolam Karthikeyan, Soosai Arulselvi, Esvaran Vijayagowri, Kalipatty Nalliappan Ganesan, Vaikuntavasan Paranidharan, Sudha K. Nair, Raman Babu, Jegadeesan Ramalingam, Muthurajan Raveendran, Natesan Senthil

**Affiliations:** ^1^ Department of Plant Biotechnology, Centre for Plant Molecular Biology and Biotechnology, Tamil Nadu Agricultural University, Coimbatore, India; ^2^ Asian Regional Maize Program, International Maize and Wheat Improvement Center (CIMMYT), ICRISAT Campus, Patancheru, India; ^3^ Department of Crop Improvement, Kumaraguru Institute of Agriculture, Erode, India; ^4^ Department of Millets, Centre for Plant Breeding and Genetics, Tamil Nadu Agricultural University, Coimbatore, India; ^5^ Department of Plant Breeding and Genetics, Agricultural College and Research Institute, Tamil Nadu Agricultural University, Madurai, India; ^6^ Department of Biotechnology, Centre of Innovation, Agricultural College and Research Institute, Tamil Nadu Agricultural University, Madurai, India; ^7^ Agricultural College and Research Institute, Thanjavur, Tamil Nadu Agricultural University, Thanjavur, India; ^8^ Department of Forage Crops, Centre for Plant Breeding and Genetics, Tamil Nadu Agricultural University, Coimbatore, India; ^9^ Department of Plant Pathology, Centre for Plant Protection Studies, Tamil Nadu Agricultural University, Coimbatore, India; ^10^ Corteva Agrisciences, Multi Crop Research Centre, Hyderabad, India; ^11^ Department of Plant Molecular Biology and Bioinformatics, Centre for Plant Molecular Biology and Biotechnology, Tamil Nadu Agricultural University, Coimbatore, India

**Keywords:** genotyping by sequencing, maize, single nucleotide polymorphism, sorghum downy mildew, QTL

## Abstract

Sorghum downy mildew (SDM), caused by the biotrophic fungi *Peronosclerospora sorghi* *,* threatens maize production worldwide, including India. To identify quantitative trait loci (QTL) associated with resistance to SDM, we used a recombinant inbred line (RIL) population derived from a cross between resistant inbred line UMI936 (w) and susceptible inbred line UMI79. The RIL population was phenotyped for SDM resistance in three environments [E1-field (Coimbatore), E2-greenhouse (Coimbatore), and E3-field (Mandya)] and also utilized to construct the genetic linkage map by genotyping by sequencing (GBS) approach. The map comprises 1516 SNP markers in 10 linkage groups (LGs) with a total length of 6924.7 cM and an average marker distance of 4.57 cM. The QTL analysis with the phenotype and marker data detected nine QTL on chromosome 1, 2, 3, 5, 6, and 7 across three environments. Of these, QTL namely *qDMR1.2, qDMR3.1*, *qDMR5.1*, and *qDMR6.1* were notable due to their high phenotypic variance. *qDMR3.1* from chromosome 3 was detected in more than one environment (E1 and E2), explaining the 10.3% and 13.1% phenotypic variance. Three QTL, *qDMR1.2, qDMR5.1*, and *qDMR6.1* from chromosomes 1, 5, and 6 were identified in either E1 or E3, explaining 15.2%–18% phenotypic variance. Moreover, genome mining on three QTL (*qDMR3.1*, *qDMR5.1*, and *qDMR6.1*) reveals the putative candidate genes related to SDM resistance. The information generated in this study will be helpful for map-based cloning and marker-assisted selection in maize breeding programs.

## Introduction

Maize (*Zea mays* L.) is a staple crop cultivated in tropical and temperate regions worldwide and provides nutrient-rich foods to billions of people. In recent years, annual maize production has declined due to biotic and abiotic stresses (Yadav et al., 2015). Among the biotic stresses, sorghum downy mildew (SDM) is one of the important diseases caused by the fungal pathogen *Peronosclerospora sorghi* in several tropical Asian countries. SDM is predominant in Indian states, including Andhra Pradesh, Tamil Nadu, and Karnataka, where 30–40% yield losses have been reported (Krishnappa et al., 1995). SDM mainly infects the crop at the seedling emergence stage *via* soil-borne oospores or wind-driven conidia (Bock et al., 1998). The infected young plants usually die early, while later-stage infection produces localized lesions that perpetuate systemically and produce unfertile plants (Schuh et al., 1986). The seed treatment and concomitant foliar sprays of metalaxyl controlled SDM disease development, but in recent times, chemical resistance in *P. sorghi* has been reported (Isakeit and Jaster, 2005; Rashid et al., 2018). Hence, the development of resistant cultivars is a priority for maize breeders to meet the future demands of maize production. Breeding for disease resistance is continued to be a challenge due to the complexity of the disease resistance mechanisms. Therefore, genomics approaches coupled with breeding methods are being used to investigate the genetic basis of disease resistance.

Maize genomics research has advanced due to the availability of whole-genome sequence (WGS) data. Thousands of molecular markers (Sharopova et al., 2002; Wang et al., 2011; Qu and Liu, 2013; Xu et al., 2017; Tian et al., 2021) and several genetic linkage maps (Pan et al., 2012; Babu et al., 2015; Su et al., 2017; Fenton et al., 2018; Xie et al., 2019) have been developed and used to identify the quantitative trait loci (QTL) associated to agronomically important traits and SDM resistance in maize (Nair et al., 2005; Lohithaswa et al., 2015; Zhao et al., 2018; Kim et al., 2020). To date, several SDM resistance-associated QTL have been located on chromosome 1, 2, 3, 6, 7, 9, and 10 using SSR and/or RFLP markers based on linkage maps. George et al. (2003) evaluated Ki3×CML139 derived recombinant inbred lines (RILs) for five diverse downy mildews of Asia and mapped six QTL on the chromosome 1, 2, 6, 7, and 10. Studies by Nair et al. (2005) identified two major QTL, each on the chromosome 3 and 6 and a minor QTL on chromosome 2 for SDM resistance in the CM139 × NAI116 derived backcross population. Sabry et al. (2006) detected a major QTL on chromosome 2, and two minor QTL on chromosome 3 and 9 in the F_3_ population screened for *P. sorghi* in Thailand, United States, and Egypt. Further, nine putative SDM resistant loci were identified by Jampatong et al. (2013) on six chromosomes (2, 3, 4, 5, 6, and 9) and three loci by Lohithaswa et al. (2015) on the chromosome of 2, 3, and 6. A previous study in our research group by Jadhav et al. (2019) localized the QTL for SDM resistance on chromosome 3 and 6 in the same region as that of Nair et al. (2005). Recently, Kim et al. (2020) used the map consisting of 691 SSR and 36 RFLP markers with an average inter-marker interval of 9.12 cM to map a major QTL on chromosome 2 for SDM resistance in B73 × Ki11 derived RIL population.

Genetic linkage maps constructed using the low-density markers have numerous large uncovered spaces, restricting gene/QTL mapping (Chen et al., 2016). The abundant and non-clustered distribution of single nucleotide polymorphisms (SNP) in the genome has better advantages and reliability. The automation in SNP detection has given SNP markers an edge over other marker systems. Besides, the remarkable advances in high throughput sequencing technologies like next-generation sequencing (NGS) and computational tools facilitate the construction of highest resolution genetic maps (Yan et al., 2010; Karthikeyan et al., 2017). Nagabhushan et al. (2017) identified three putative QTL (chromosomes 2, 3, and 6) that control SDM resistance in the F_2_:_3_ population using 128 SSR and 191 SNP markers. The average inter-marker distance was reduced to 6.47 cM relative to the genetic maps constructed with traditional SSR and/or RFLP markers. However, to our knowledge, the average inter-marker distance is still more than 5 cM in all published articles for SDM disease in maize. Hence, there is a broader scope for constructing dense maps and, if possible, the subsequent refinement of target locus for SDM resistance. In regard to the available literature information, a high-density SNP map has yet to be used to map the QTL for SDM resistance in maize. In recent days, genotyping by sequencing (GBS) has generated a large number of SNPs, which are used in the construction of high-resolution genetic linkage maps and QTL analysis. It has been successful in the detection of QTL associated with resistance for Mediterranean corn borer (Jiménez-Galindo et al., 2017), fusarium ear rot (Maschietto et al., 2017), and grey leaf spot (Du et al., 2020) in maize.

Taking into account the above, the objectives of this research were to: 1) evaluate the RIL population for SDM resistance, 2) construct the high-density linkage map to detect the QTL associated with SDM resistance; 3) trace the possible candidate genes related to SDM resistance.

## Materials and Methods

### Plant Materials and Experimental Sites

The RIL population comprised 150 lines from the cross between SDM susceptible inbred, UMI79 and resistant inbred, UMI936 (w) was used in this study. The single-seed descent (SSD) method was followed to develop the RIL population. The SDM disease screening was carried out in three environments, namely Experimental farm, Department of Millets, Centre for Plant Breeding and Genetics, Tamil Nadu Agricultural University, Coimbatore, Tamil Nadu, India (Environment 1, E1_2013), Glasshouse, Centre for Plant Molecular Biology, TNAU, Coimbatore, Tamil Nadu, India (Environment 2, E2_2013) and Zonal Agriculture Research Station, V.C. Farm, Mandya, Karnataka, India (Environment 3, E3_2015).

### Phenotyping of SDM Resistance

During the field screening at E1 and E3 environments, the RIL population and their parents were grown in alpha-lattice design with two replications by growing in 3-m-long rows with row-to-row spacing of 75 cm and plant-to-plant spacing of 20 cm. The artificial epiphytotic conditions were created using the “spreader row technique” in the field (Craig et al., 1977; Cardwell et al., 1997). Three weeks before sowing of the test entry (RILs), highly susceptible cultivar (CM-500), was sown along the entire border of the experimental block and in dense stands after every eight rows to function as a spreader row. At the second or third leaf stage, the conidial spraying was adopted on the spreader row by the following process, in the early morning, the SDM-infected leaves were collected from downy mildew nursery, washed in water, and a conidial suspension was prepared. The spraying was continued for a week. When sufficient indications of infection occurred on the spreader rows, test entries were planted alongside with susceptible controls, and a similar spraying operation was performed for 1 week on the test entries to maintain consistent disease pressure and prevent disease escape. Disease screening at glasshouse (E2) was done by adopting the standard method “Seedling spray inoculation technique” (Craig et al., 1977; Narayana et al., 1995). Two replications for each test line were performed and plants were maintained according to the method adopted by Sabry et al., 2006. Temperature and humidity were maintained at 25 ± 2°C and 80–90% respectively in favour of disease development.

The total number of infected plants to the total number of plants in each test entry was counted and expressed in percentage. Based on the percentage of disease incidence, the entries were categorized as resistant (0%–10%), moderately resistant (>10%–30%), moderately susceptible (>30%–50%), and susceptible (>50%) (Lal and Singh, 1984).

### DNA Isolation and Genotyping by Sequencing

Genomic DNA was isolated from RIL population and the parental lines following the standard CIMMYT laboratory protocol (CIMMYT, 2005). The isolated DNA was checked for quality and quantity using a NanoDrop ND-1000 (Thermo Fisher Scientific Inc., United States). The GBS libraries were constructed in 96-plex and sequenced on Illumina HiSeq 2000 at the Institute of Genomic Diversity, Cornell University, Ithaca, United States. In order to identify SNPs, the sequence data of parental lines and RILs from raw FASTQ files were analyzed in the TASSEL-GBS pipeline with B73 maize reference genome _v2 (https://www.maizegdb.org/). The call rate (CR) ≥0.9 and minor allele frequency (MAF) ≥0.4 were used for filtering in 8k SNPs. SNP markers were filtered for monomorphic, heterozygous SNPs, and the input file was prepared for QTL mapping.

### Genetic Map Construction and QTL Analysis

A set of 150 RILs was sequenced with GBS, but three lines were removed due to more than 10% non-parental data points, and only 147 RILs were utilized for linkage map construction. The SNP data in an allele-based format (i.e., A, T, G, or C) was converted to parent-based ABH format by utilizing the SNP functionality of IciMapping v4.2.53. Binning was performed to filter out redundant and highly distorted markers (*p* < 0.001). To construct the linkage map, grouping was done based on anchor information, and ordering of the markers within each chromosome was carried out based on the physical position. Recombination distance was estimated in centimorgan (cM) with kosambi mapping function (Kosambi, 1944), and the input file for QTL analysis was also generated. For detection of QTL conferring SDM resistance, the transformed phenotypic data mean values of three environments (E1, E2, and E3) were analyzed with inclusive composite interval mapping method using IciMapping v4.2.53 software (Li et al., 2015) with a 1 cM scanning step. The highest *p*-value for variables entered in stepwise phenotypic on marker variables (PIN) was set at 0.001. A LOD threshold of 2.5 was applied to identify the more likely position of QTL. The QTL was denoted as *qDMR2.1* where *qDMR* corresponds to the genomic region associated with SDM resistance and 2.1 represents the first QTL identified on chromosome 2.

### Mining of Candidate Genes in the QTL Regions

The QTL with high phenotypic variance explained (>10%) were considered as major QTL and selected to tag the putative candidate gens for SDM resistance. The physical distance (kb) of the QTL among the two flanking markers was detected by combining the genetic linkage map and physical map of B73 maize reference genome_v2 (https://www.maizegdb.org/accessed on 26th January 2022). Moreover, the information of existing gene models around the major QTL genomic intervals (Including up and downstream of 250 kb) was collected from the B73 maize reference genome_v2. The possible candidate genes of SDM resistance were searched according to their involvement in disease resistance with the support of available literature knowledge.

### Statistical Analysis

The arc-sine transformation was performed in the phenotypic data to approximate the normality in trait distribution. Broad sense heritability for SDM disease incidence was calculated using the formula ( $H^{2}=\sigma^{2}g/\left(\sigma^{2}g+\sigma^{2}e/r\right)$ ) where $\sigma^{2}g$ is the genotypic variance, $\sigma^{2}e$ is the variance due to environmental factors, and r is the number of replications.

## Results

### Phenotypic Variation of SDM Resistance

**FIGURE 1:**
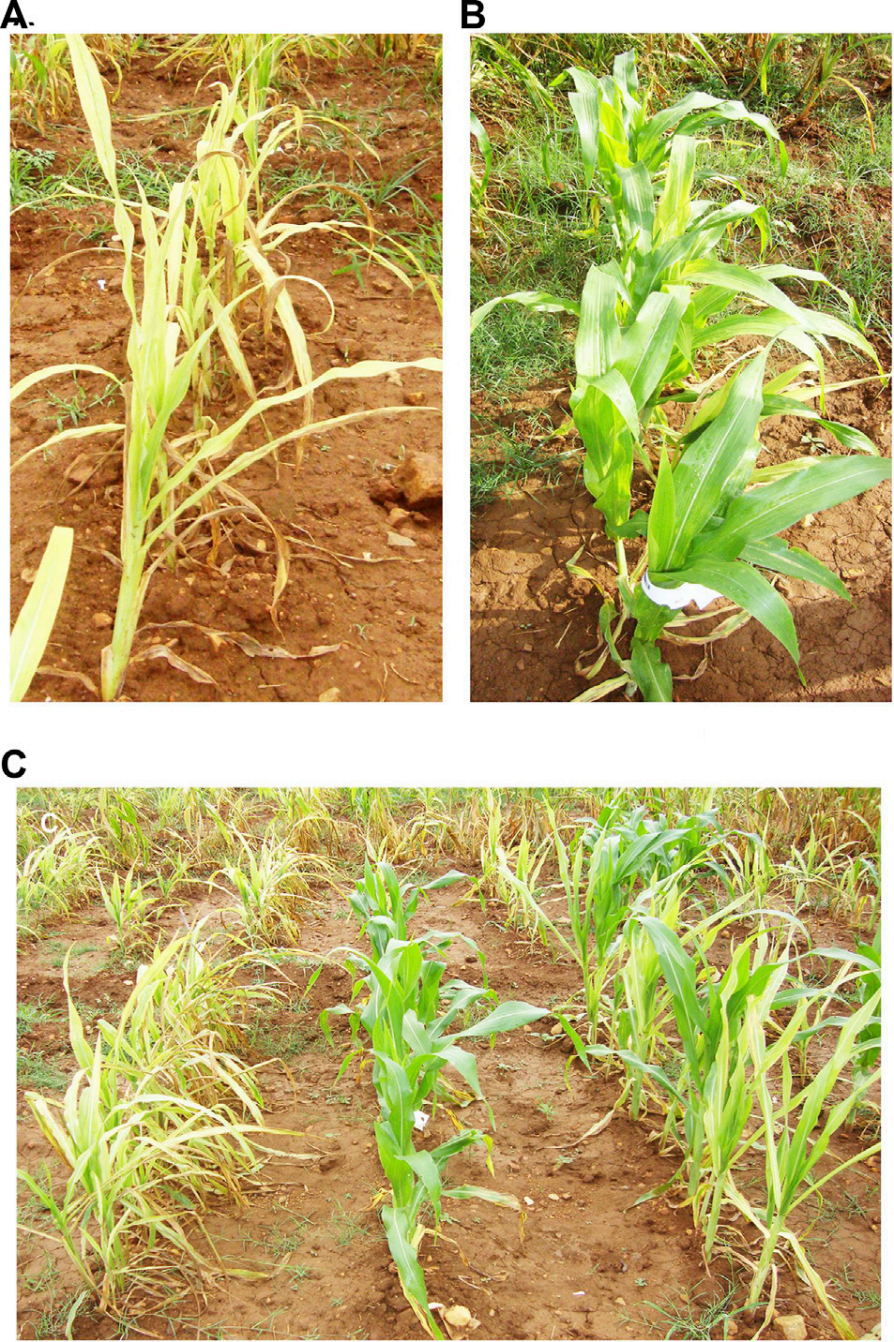
**(A)** UMI79 [Susceptible], **(B)** UMI936 (w) [Resistant], and **(C)** recombinant inbred line (RIL) population response to sorghum downy mildew.

A RIL population and the parental lines were characterized for SDM disease reaction in three different environments (E1, E2, and E3). The descriptive statistics data is provided in Table 1. The parental lines, UMI936 (w) (<10% mean disease score) and UMI79 (>95% mean disease score), exhibited contrast phenotypes consistently while the RILs registered varying levels of disease response at the test environments. The typical disease symptoms of SDM observed in the screening plot are depicted in Figure 1. At E1, disease incidence varied from 7.0 to 94.7%, from 6.7 to 93.0% in the E2, whereas it differed between 28.9 and 100% at E3. The RIL population showed non-normal distribution in all the environments with the disease response skewed towards resistant parent at E1 and E2 and towards susceptible parent at E3. The broad sense of heritability (H^2^) was high in all the three environments, i.e., 96.70%, 97.00%, and 87.50% at E1, E2, and E3, respectively. Thus, using the phenotype data of SDM incidence for QTL analysis is meaningful.

**TABLE 1:** Descriptive statistics and heritability on sorghum downy mildew disease incidence in recombinant inbred line (RIL) population derived from a cross of UMI79 × UMI936 (w) over the three environments.

S. No.	Environment	UMI79	UMI936(w)	Recombinant inbred lines		
		Mean ± SD^a^	Mean ± SD^a^	Range	Mean ± SD^a^	H^b^ (%)
1	E1_Field (Coimbatore)	100.0 ± 0.00	7.70 ± 0.80	7.00–94.70	26.19 ± 21.93	96.70
2	E2_Glass house (Coimbatore)	96.67 ± 4.72	8.01 ± 0.45	6.70–93.00	26.79 ± 23.24	97.00
3	E3_ Field (Mandya)	100.0 ± 0.00	25.60 ± 3.20	28.90–100.00	86.55 ± 13.21	87.45

a Standard deviation.

b Broad sense heritability.

### Population Sequencing and Identification of SNPs

**FIGURE 2:**
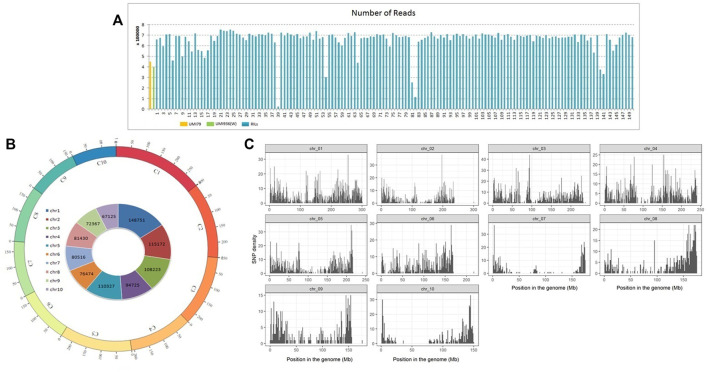
Number of reads and genome wide distribution of SNPs in recombinant inbred line (RIL) population of UMI79 × UMI936 (w). Note; **(A)** Number of reads generated per sample in UMI79, UMI936 (w), and RIL population, **(B)** SNPs in maize Chromosomes, labeled as C1 to C10 and each chromosome is shown in a different colour. The numbers on arches represent the scale for the size of chromosomes in Mb. The numbers on inside circle represents the total number of RAW SNPs in each chromosome, **(C)** Distribution of filtered high quality SNPs in maize chromosomes.

A total of 18.6 Gb (203,115,257) GBS reads including 447,545 reads from UMI79 and 398,099 reads from UMI936 (w) were generated using HiSeq2000 platform. Individual RIL reads ranged from 0.02 million to 0.75 million, with an average of 0.66 million reads (Figure 2). The barcode and linker bases were removed from each sample read before the RE remnants were removed. Subsequent SNP calling in TASSEL-GBS pipeline identified 955,110 raw SNPs which varied from 67,125 on chromosome 10 to 148,751 SNPs on chromosome 1 (Figure 2). Based on the twin criterion of call rate (>0.9) and minor allele frequency (MAF>0.4), 8,816 SNPs were selected (Figure 2). A total of 6,381 SNPs were found to be polymorphic between parental lines. Of these, 2,129 SNPs were homozygous for both parental lines and used for further analysis. A total of 2,101 SNPs were retained after removing those with a missing data rate per site >30% for downstream analyses. Redundant and segregation distorted markers were filtered by binning. Among 2,101 high-quality SNPs, 1516 SNPs (72.16%) showed a normal Mendelian segregation ratio of 1:1, whereas 585 SNPs (27.84%) significantly deviated from the Mendelian segregation. As a result, 1516 SNP markers were selected for linkage map construction.

### SNP Based Genetic Linkage Map

**FIGURE 3:**
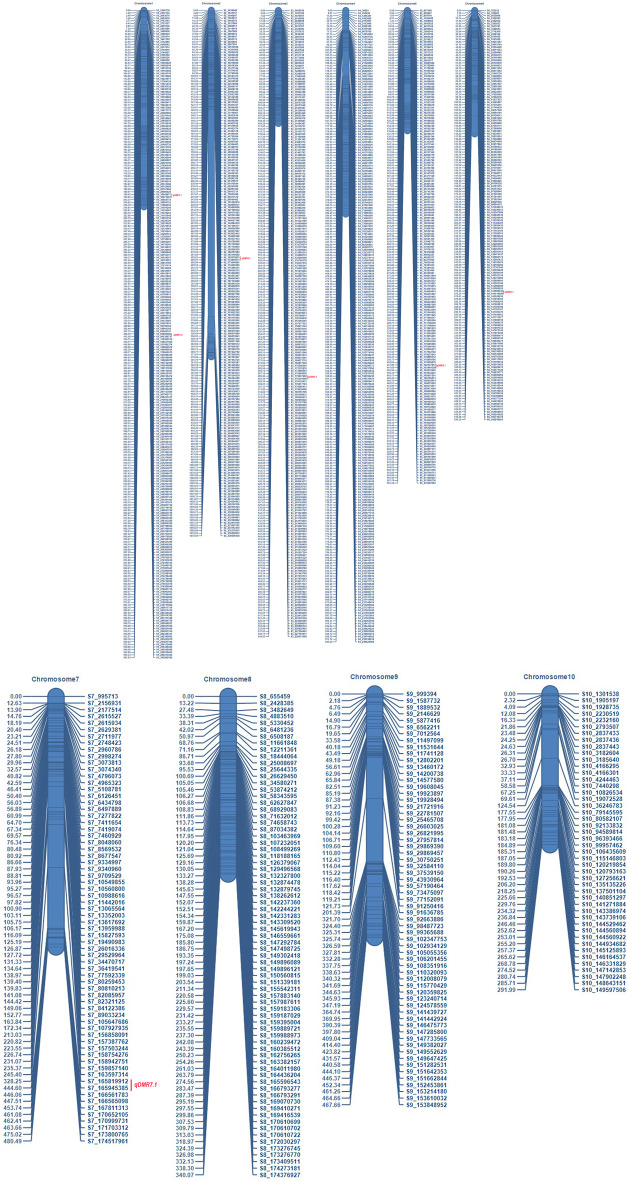
Genetic linkage map of recombinant inbred line (RIL) population developed from the cross of UMI79×UMI936 (w) Note: For each chromosome, the SNP markers were shown on right side, and the genetic distances in cM on the left side. QTL are depicted on the right side with red color.

The number of SNPs has decreased from millions to thousands due to the stringent selection criteria. Consequently, a linkage map was constructed from the data of 1516 SNP markers (Figure 3). The marker density was highest in chromosome 1 (247 SNPs) and lowest in chromosome 10 (46 SNPs). The map spanned a total length of 6924.7 cM across the whole genome with a marker interval of on average of 4.57 cM. The map length varied from 292 cM (chromosome 10) to 1678.5 cM for chromosome 2. The average inter-marker distance was highest for chromosome 2 (8.35 cM) and lowest for chromosome 3 (2.29 cM). The characteristics of the linkage map are presented in Table 2.

**TABLE 2:** Characteristics of genetic linkage map constructed using recombinant inbred line (RIL) population derived from a cross of UMI79 × UMI936 (w).

S.No	Chromosome	SNPs identified	Filtered SNPs	Mapped SNPs	SNPs mapped (%)	Map length (cM)	Average marker interval (cM)
1	1	148751	327	247	75.54	951.0	3.85
2	2	115172	277	201	72.56	1678.5	8.35
3	3	108223	331	239	72.21	546.8	2.29
4	4	94725	370	241	65.14	985.4	4.08
5	5	110327	233	181	77.68	583.3	3.22
6	6	76474	209	157	75.12	599.4	3.82
7	7	80516	96	68	70.83	480.5	7.06
8	8	81430	88	73	82.95	340.1	4.66
9	9	72367	105	63	60.00	467.7	7.42
10	10	67125	65	46	70.77	292.0	6.35
	Total	955110	2101	1516	72.16	6924.7	4.57
	Average	95511	210.1	151.6	72.16	692.47	4.57

### QTL for SDM Resistance

To identify QTL for SDM resistance, a single-environment QTL analysis was performed with the phenotype data of three environments and 1516 SNP markers. A total of nine QTL were detected on six chromosomes (1, 2, 3, 5, 6, and 7) across the three environments (Table 3). Based on the percentage phenotypic variance explained by the QTL (PVE), the QTL were classified as major effect QTL (>10%) and minor effect QTL (<10%). LOD scores and PVE values for the detected QTL ranged from 2.6 to 5.1 and 8.2–18.0%, respectively. The additive effects varied from −5.1 to 7.1, which indicates that the favorable alleles for SDM resistance were contributed by both parental lines. At field condition E1, two major QTL on chromosome 3 and 5 and one minor QTL on chromosome 2 were detected. The major effect QTL (*qDMR5.1*) mapped on chromosome 5 between the markers S5_182137917 and S5_183484329 explained 15.2% of the phenotypic variance with an additive effect of 6.0. Another major effect of QTL (*qDMR3.1*) on chromosome 3 with a LOD score of 4.8 flanked by the markers S3_175937292 and S3_176301690 explained 10.3% of phenotypic variation. The resistant parent, UMI936 (w), contributed the favourable alleles for *qDMR5.1* and *qDMR3.1*. The minor effect QTL, designated as *qDMR2.1*, was identified on chromosome 2 between the markers S2_163678815 and S2_172849073 with a LOD score and phenotypic variation of 3.8 and 8.2% respectively. The susceptible parent, UMI79, contributed the favourable alleles for this QTL. At glasshouse condition E2, major QTL (*qDMR3.1*) and minor QTL (*qDMR2.1*) QTL with 13.1% and 8.7% PVE were detected. These two QTL were also detected in the E1. At field condition E3, four QTL were identified on chromosome 1, 6, and 7. The major effect QTL, designated as *qDMR1.2*, was localized on chromosome 1 between the S1_85896686 and S1_99538872, explaining 16.6% phenotypic variance. Another QTL denoted as *qDMR6.1*, which explained 18% phenotypic variation, was mapped between the markers S6_143989532 and S6_144119580 on chromosome 6. For both QTL, the favourable alleles were contributed by the resistant parent, i.e., UMI936 (w). The minor QTL designated as *qDMR1.1* and *qDMR7.1* were flanked by the markers S1_30042877-S1_30571407 and S7_165819912-S7_165945385, respectively. The favourable alleles of these minor QTL were contributed by the susceptible parent.

**TABLE 3:** Details of QTL identified for SDM resistance in the recombinant inbred line (RIL) population derived from a cross of UMI79 × UMI936 (w) over the three environments.

S.No	Environment	QTL	Chr^a^	Position (cM)	LOD^b^	PVE^c^ (%)	Add^d^	CI^e^ (cM)	QTL interval		QTL size (Mb)
									Left marker	Right marker	
1	E1_Field (Coimbatore)	qDMR2.1	2	283	3.8	8.2	−4.4	281.5–283.5	S2_163678815	S2_172849073	9.17
		qDMR3.1	3	292	4.8	10.3	4.9	289.5–295.5	S3_175937292	S3_176301690	0.36
		qDMR5.1	5	450	2.9	15.2	6.0	449.5–451.5	S5_182137917	S5_183484329	1.35
2	E2_Glass house (Coimbatore)	qDMR2.1	2	283	3.2	8.7	−4.5	281.5–284.5	S2_163678815	S2_172849073	9.17
		qDMR3.1	3	292	4.2	13.1	5.1	289.5–295.5	S3_175937292	S3_176301690	0.36
3	E3_ Field (Mandya)	qDMR1.1	1	194	2.5	8.8	−4.9	189.5–200.5	S1_30042877	S1_30571407	0.53
		qDMR1.2	1	407	3.1	16.6	6.7	404.5–408.5	S1_85896686	S1_99538872	13.64
		qDMR6.1	6	271	5.1	18.0	7.1	270.5–272.5	S6_143989532	S6_144119580	0.13
		qDMR7.1	7	444	2.6	8.9	−5.1	421.5–446.5	S7_165819912	S7_165945385	0.13

a Chromosome.

b The log of odds (LOD) value at the peak likelihood of the QTL.

c Phenotypic variances (%) explained by the QTL.

d Positive additive effect indicates the contribution of the allele from UMI936 (w) and negative additive effect indicates contribution of the allele from UMI79).

e Confidence intervals.

### Tracing the Candidate Genes for SDM Resistance in the Selected QTL

Among the nine QTL, we have selected major QTL (*qDMR3.1*, *qDMR5.1,* and *qDMR6.1*) for searching the candidate genes to SDM resistance. Although the QTL *qDMR1.2* on chromosome 1 showed a high phenotypic variance, further investigation was not pursued due to the large genomic interval. To select the putative candidate genes for SDM-resistance, we searched the genes around the corresponding genomic region of *qDMR3.1* on chromosome 3, *qDMR5.1* on chromosome 5*,* and *qDMR6.1* on chromosome 6*.* According to the maize reference genome B73, a total of 26, 30, and 18 genes existed in the *qDMR3.1*, *qDMR5.1*, and *qDMR6.1* genomic regions, respectively (Supplementary Tables S1–S3). Further, to pick the possible candidate genes, we exploited the annotated details of these genes. It revealed several putative candidate genes that are directly or indirectly related to defense response to plants’ environmental stress. They belong to the different gene families such as NDR1/HIN1-like protein 10, Spotted leaf 11/ARM repeat superfamily protein, glycine-rich protein, protein ligase plant U-box 22, RING/U-box superfamily protein, endochitinase precursor4, and a cysteine-rich RLK (RECEPTOR-like protein kinase) 2. The details of the candidate genes are listed in Table 4.

**TABLE 4:** Most likely candidates for *qDMR3.1*, *qDMR5.1*, and *qDMR6.1* on chromosome 3, 5, and 6

S. No.	Gene ID	Chr^a^	Physical position (bp)^b^	Annotation
1	GRMZM2G401009	3	175936709	RING/U-box superfamily protein
2	GRMZM2G135703	3	175965418	C2C2-Dof-transcription factor 19
3	GRMZM2G135706	3	175974833	Spotted leaf 11/ARM repeat superfamily protein
4	GRMZM2G135713	3	175987501	Protein ligase plant U-box 22
8	GRMZM2G074248	3	176301626	NDR1/HIN1-like protein 10
9	GRMZM2G074236	3	176302928	NDR1/HIN1-like protein 10
10	GRMZM5G832473	3	176510723	NAC domain containing protein 90
10	GRMZM2G300771	5	182032083	cGMP-dependent protein kinase, isozyme 2 forms cD4/T1/T3A
11	GRMZM2G137426	5	182032083	basic helix-loop-helix (bHLH) DNA-binding superfamily protein
11	GRMZM2G137409	5	182124005	4-hydroxy-3-methylbut-2-enyl diphosphate synthase/lemon white2
12	GRMZM2G129189	5	182518442	Endochitinase precursor4
13	GRMZM2G300812	5	182577369	C2 domain-containing protein/Gram domain-containing protein
14	GRMZM2G007466	5	183121089	Integrin-linked protein kinase family
15	GRMZM2G021777	5	183367991	CONSTANS-like 4
16	GRMZM2G027105	5	183428462	RNA recognition motif and CCHC-type zinc finger domains containing protein
17	GRMZM5G850997	5	183703994	RING/U-box superfamily protein
15	GRMZM5G869788	6	143956525	Glycosyltransferase family 61 protein
16	GRMZM2G424582	6	143982360	Protein kinases; ubiquitin-protein ligases
17	GRMZM2G427337	6	144117824	Glycine-rich protein
18	GRMZM2G332107	6	144413748	Cysteine-rich RLK (Receptor-like protein kinase) 2

a Chromosome.

b Gene start position, Note; the physical postion and gene details accessed on maize GDB (https://www.maizegdb.org/) (Verified 26th January 2022).

## Discussion

### SDM Resistance in Maize

Understanding the genetics of resistance and the role of genes in developing resistance or susceptibility will be necessary for the maize breeders aiming to develop the cultivar’s resistance to SDM. In this study, we used the RIL population derived from the cross between UMI79 (susceptible) and UMI936 (w) (resistant) to characterize the SDM resistance. The population screened for SDM resistance in field and/or glasshouse conditions at three environments (E1, E2, and E3). The phenotypic means for SDM disease showed skewed distribution in the RIL population. This deviation from normality is more common for diseases including SDM, and most of the works published so far reported atypical distribution for SDM disease reaction (George et al., 2003; Nair et al., 2005; Phumichai et al., 2012; Lohithaswa et al., 2015). The phenotypic distribution leaned towards resistance at E1 and E2 and towards susceptibility at E3. The higher mean percent disease incidence of 86.6 at the environment of E3 relative to 26.19 (E1) and 26.79 (E2) indicated the prevalence of high disease pressure or more virulent strain of *P. sorghi* at E3. Similar observations were recorded for SDM by George et al. (2003), where the RILs were skewed towards susceptibility due to high disease pressure while towards resistance due to low disease pressure. Higher heritability (87.5–97.0%) in all three environments explained the presence of relatively high genetic variance for SDM resistance. Such higher estimates of heritability for quantitative resistance were observed in many host-pathogen systems (Keller et al., 2000), including SDM resistance (Nair et al., 2005; Jampatong et al., 2013).

### Refining the Genomic Region for SDM Resistance by Improved Genetic Map

Detecting QTL needs a high-resolution linkage map with minimum average marker interval. Our previous map constructed using the RIL population had only 58 SSR markers, and the marker density was very low. The present map comprises high-quality 1516 SNP markers and has a higher marker density than the earlier map (Jadhav et al., 2019). According to the available literature, no other previous SDM research in maize has ever achieved the current map marker density and genome coverage. This map covered the entire genome with a total length of 6924.7 cM and an average marker interval of 4.57 cM. The linkage map expansion is most likely due to the segregation distortion, heterozygosity, Allele switching, excessive single cross events, and unexpected double recombinants like genotyping errors. The average inter-marker distance was highest on chromosome 2 (8.35 cM) and lowest on chromosome 3 (2.29 cM). In this study, we used a high criterion to SNP call rate and sample call rate to avoid the genotyping errors. Although the filtering steps eliminated most of the SNPs, the remaining markers and samples had high-quality scores that ensured the construction of genetic map with a high level of accuracy. The adopted stringent criteria reduced the number of high quality SNP markers and also increased the inter marker distance and map length; however, this is very common in GBS data. Every 1% error rate in a marker adds about 2 cM to the linkage map (Cartwright et al., 2007; Truong et al., 2014). QTL study using 1516 SNP markers and phenotypic data of three different environments were revealed nine QTL for SDM resistance in six of the ten chromosomes (1, 2, 3, 5, 6, and 7). Among them, *qDMR2.1* and *qDMR3.1* were located in the marker intervals S2_163678815 - S2_172849073 and S3_175937292 - S3_176301690, respectively, were consistently detected in more than one environment (E1 and E2) and regarded as stable QTL. Whereas other QTL, *qDMR5.1* was localized on chromosome 5 between the markers S5_182137917 and S5_183484329, *qDMR1.2* was localized on chromosome 1 between the markers S1_85896686 and S1_99538872, *qDMR6.1* was localized on chromosome 6 between the markers S6_143989532 and S6_144119580, *qDMR1.1* was localized on chromosome 1 between the markers S1_30042877- S1_30571407 and *qDMR7.1* were localized on chromosome 7 between the markers S7_165819912-S7_165945385 were detected only in a single environment either E1 or E3. These QTL explained the phenotypic variance ranging from 8.2 to 18.0%, and QTL regions were delimited to a genomic interval of approximately ranging from 0.13 to 13.64 Mb. Moreover, the QTL identified in this study specific to the environment, the reason behind that was resistance QTL can sometimes only be detected under certain environmental conditions (soil, climate, pathogen population), or in specific genetic backgrounds or cross-type (George et al., 2003; Nagabhushan et al., 2017). In summary, we have detected nine QTL associated with SDM resistance over the 2 years and their precise chromosomal locations using the improved genetic map. It helps to understand the genetic architecture of SDM resistance and is also helpful to explore the genes for SDM resistance.

### Comparison of the QTL With the Already Reported QTL of SDM Resistance in Maize

**FIGURE 4:**
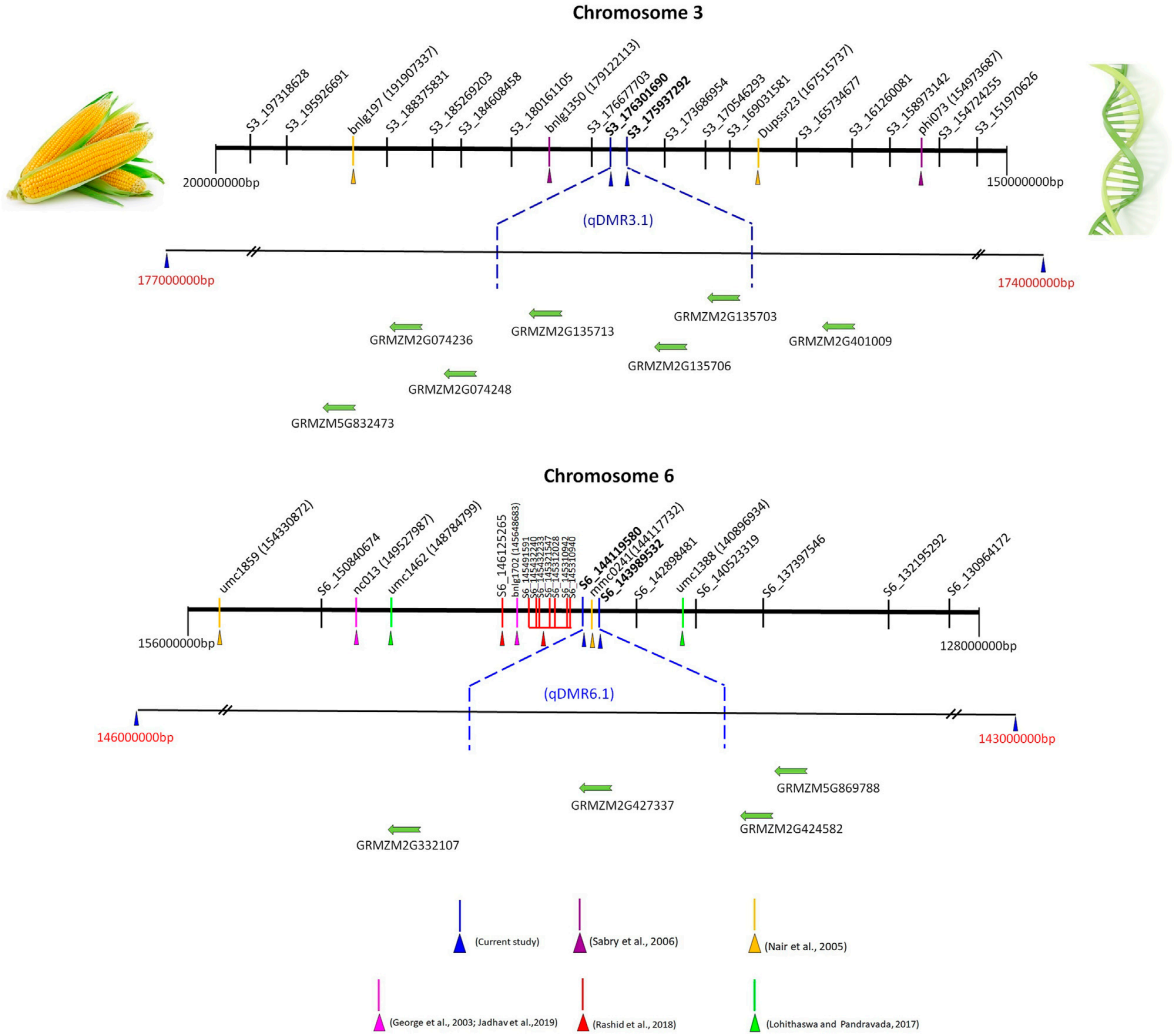
Integrative views of qDMR3.1 and qDMR6.1 genomic region (s) with already reported SDM resistance QTL located on chromosome 3 and 6. Note: The numbers near to the SSR markers (phi073, Dupssr23, bnlg1350, and bnlg197 at chromosome 3 and nc013, bnlg1702, mmc0241, umc 1859, umc1388, and umc1462 at chromosome 6) in bracket are physical positions (bp) of markers in maize genome, while SNP markers ID denote the chromosome number and physical position. The physical position and gene details accessed on maize GDB (https://www.maizegdb.org/) (Verified 26th January 2022). SDM resistance QTL and linked markers [phi073, Dupssr23, bnlg1350, and bnlg197] on chromosome 3 (Nair et al., 2005; Sabry et al., 2006). SDM resistance QTL and linked markers [nc013, bnlg1702, mmc0241, umc 1859, umc1388, umc1462, S6_145310940, S6_145310942, S6_145312028, S6_145321547, S6_145432233, S6_145432240, S6_145491591, and S6_146125265] on chromosome 6 (George et al., 2003; Nair et al., 2005; Nagabhushan et al., 2017; Rashid et al., 2018; Jadhav et al., 2019). The putative candidate genes for qDMR3.1 and qDMR6.1 genomic region (s) only presented in this figure, the complete gene details around the QTL region summarized in Supplementary Tables S1–3. The physical distance between markers or genes is approximate.

To date, several QTL have been reported for SDM resistance on the chromosome 2, 3, and 6 (George et al., 2003; Nair et al., 2005; Jampatong et al., 2013; Lohithaswa et al., 2015; Nagabhushan et al., 2017). We have compared the nine QTL (over the three environments) detected in the present study with the published QTL linked to SDM resistance based on the marker’s physical position in the reference genome B73 RefGen_V2 sequence. *qDMR1.2*, *qDMR3.1*, *qDMR5.1*, and *qDMR6.1* were identified to be major QTL with PVE ranging from 10.3 to 18.0%. Notably, a favorable allele was contributed by resistant parent UMI936 (w) for all these QTL. *qDMR1.2*, a QTL identified on chromosome 1 at 85.9—99.5 Mb region, significantly impacted SDM resistance in the field at E3 (PVE = 16.6%). These findings resembled Rashid et al. (2018), who discovered an SNP marker associated with SDM resistance at the nucleotide position 88.9 Mb through association mapping. Another QTL identified on chromosome 3, denoted as *qDMR3.1*, had major effects and was consistently detected in more than one environment (E1 and E2) (Figure 4). It was located at a physical distance of 175.9–176.3 Mb. This region overlapped with the QTL flanked by markers phi073-bnlg1350 which are positioned at 154.97–179.12 Mb on the chromosomal bin 3.04–3.05 (Sabry et al., 2006) and QTL flanked by markers Dupssr23-bnlg197 positioned at 167.51—191.90 Mb region on chromosomal bin 3.05 (Nair et al., 2005). We also identified the novel QTL *qDMR5.1* in 182.1–183.2 Mb region on the Chromosome 5 with major effect (PVE = 15.2%) at E1. Previously, no QTL for SDM resistance was reported around the QTL *qDMR 5.1*. Therefore, *qDMR5.1* detected in our study is considered a novel QTL. Another major QTL, *qDMR6.1*, explained the highest phenotypic variation (18%) for SDM resistance and was located on chromosome 6 at 143.98–144.12 Mb region (Figure 4). This region was determined to be intriguing since our early SSR-based map investigation detected QTL adjacent to this region with flanking markers bnlg1702 - nc013 positioned at 145.64 - 149.52 Mb (>4 Mb interval) on the physical map. The current study delimited the 4 Mb interval into 130 kb using GBS-based SNP markers. Moreover, many QTL or SNPs associated with downy mildew resistance were found in this or nearby region. Rashid et al. (2018) reported 8 SNPs associated with SDM resistance between the nucleotide positions of 145.31–146.12 Mb on chromosome 6 through association mapping. It was also consistent with the QTL discovered by George et al. (2003) (145.64–149.52 Mb) for resistance against five different downy mildew pathogens, including SDM. The QTL found by Nair et al., 2005 (144.11–154.33 Mb) and Nagabhushan et al., 2017 (140.9–148.79 Mb) on the chromosomal bin 6.05 also shared the common genomic region as the QTL discovered in our study. Three minor QTL, namely, *qDMR1.1*, *qDMR2.1*, and *qDMR7.1*, were also detected, confirming the polygenic nature of SDM resistance in maize. The favourable alleles of these QTL were contributed by the susceptible parent. Among these, *qDMR 2.1* located on chromosome 2 at a physical distance of 163.6–172.8 Mb (bin 2.06) shared the same/nearby genomic region as reported by Phumichai et al. (2012) and George et al. (2003) via association mapping and QTL mapping approach. QTL *qDMR1.1*, located on chromosome 1 at 30.04–30.57 Mb, and *qDMR 7.1*, located on chromosome 7 at 165.81—165.95 Mb regions, seems to be novel, as no QTL at the same region had previously been reported.

### Putative Candidate Genes Harbored in QTL Intervals

We chose three major QTL (*qDMR3.1*, *qDMR5.1*, and *qDMR6.1*) among the nine QTL to look for candidate genes to SDM resistance. Totally, 26, 30, and 18 genes existed around the *qDMR3.1*, *qDMR5.1*, and *qDMR6.1* genomic region on chromosome 3, 5, and 6. Further, to select the putative candidate genes, we utilized the annotation information of these genes and identified several possible candidates. *qDMR3.1* is located in a 0.36 Mb genomic interval flanked by markers S3_175937292 and S3_176301690 on chromosome 3. A total of 26 protein-coding genes existed in this genomic region. Of these, *GRMZM5G832473* encodes NAC domain-containing protein 90, and *GRMZM2G135703* encodes C2C2-Dof-transcription factor 19. Transcription factors (TFs) are crucial participants in pathogen defense. NAC TFs have been reported as positive or negative regulators of plant immunity to biotrophic, hemibiotrophic, or necrotrophic diseases, as modulators of hypersensitive responses and stomatal immunity, or as virulence targets of pathogen effectors (Yuan et al., 2019). The DNA-binding zinc finger proteins (Dofs) role in disease response to two TMV strains, PepMoV, and *Phytophthora capsici* in pepper, have been documented (Kang et al., 2016). Several Dof domain proteins related genes have been identified to alter by HSVd infection in sweet cherry (Xu et al., 2020).

*GRMZM2G135706* encodes the spotted leaf 11/ARM repeat superfamily protein. Spotted leaf11 encodes a U-box/armadillo repeat protein with E3 ubiquitin ligase activity. In rice, the spl11 mutation caused spontaneous cell death in the leaves and increased disease resistance to bacterial and fungal pathogens (Yin et al., 2000; Zeng et al., 2004). Therefore, it is reported as a negative regulator of the PCD and defense in rice. In recent years, researchers have revealed that ubiquitination is important for disease resistance in many plant species. *GRMZM2G401009* encodes RING/U-box superfamily protein and *GRMZM2G135713* encodes the plant U-box 23. In tomato (*Solanum lycopersicum*), a set of E3 ligase genes such as CMPG1 and ACRE276 identified as positive regulators of the hypersensitive response in Avr9-treated Cf-9 tobacco (*Nicotiana tabacum*) cell cultures (González-Lamothe et al., 2006; Yang et al., 2006; van den Burg et al., 2008).

Yang et al. (2006) discovered a conserved class of U-box ARMADILLO repeats E3 ligases in the Solanaceae and Brassicaceae that are positive regulators of cell death and defense. E3 ubiquitin-protein ligase has been reported to inhibit spore germination and reduce the conidial proliferation of powdery mildew pathogen on wheat (Mehta et al., 2021). Further, it mediated SAR independent resistance in Arabidopsis against DM pathogen, *Peronospora parasitica* (Kim and Delaney, 2002) and also provides innate immunity in plants. *GRMZM2G074248* and *GRMZM2G074236* are predicted to be NDR1/HIN1-like protein 10. A protein that has a similar sequence to the tobacco hairpin-induced gene (HIN1) and the Arabidopsis non-race specific disease resistance gene (NDR1). NDR1 is necessary for non-race-specific bacterial and fungal pathogen resistance. It mediates the response to systemic acquired resistance (SAR) and plays an essential role in resistance mediated by multiple R genes recognising different bacterial and oomycete pathogen isolates like avirulent *P. syringae* or *H. parasitica* (Downy mildew). *qDMR5.1* was mapped in 1.35 Mb genomic interval between S5_182137917 and S5_183484329 markers on chromosome 5, and a total of 32 protein-coding genes existed around this genomic region. Of these, several genes, including *GRMZM2G129189* (Endochitinase precursor4) (Richa et al., 2017), *GRMZM2G300812* (C2 domain-containing protein/Gram domain-containing protein) (Li et al., 2016), and *GRMZM2G007466* (Integrin-linked protein kinase family) (Brauer et al., 2016), were related with defense response to environmental stress in plants. *qDMR6.1* flanked by markers S6_143989532 and S6_144119580 located on 130 kb genomic interval at chromosome 6. A total of 20 protein-coding genes existed around this region. Of these *GRMZM2G332107* encodes cysteine-rich RLK (RECEPTOR-like protein kinase) 2. It is well understood that receptor-like kinases, specifically Cysteine (C)-rich receptor-like kinases (CRKs) are conserved upstream signaling molecules play a role in disease resistance and cell death in plants (Chen et al., 2003; Acharya et al., 2007; Ederli et al., 2011). In barley, the cysteine-rich receptor-like protein kinases role in regulating basal resistance against powdery mildew pathogen *Blumeria graminis* f. sp*. hordei* have been documented. (Rayapuram et al., 2012). *GRMZM2G424582* (Protein kinases; ubiquitin-protein ligases) (Furlan et al., 2012), and *GRMZM5G869788* (Glycosyltransferase family 61 protein) (Chowdhury et al., 2017). Collectively, this study identified several putative candidate genes to *qDMR3.1*, *qDMR5.1*, and *qDMR6.1*. However, the available information is not sufficient to determine the actual candidate gene(s) accounting for SDM resistance; additional research is required. In summary, the present study results pinpoint QTL linked to SDM resistance. There were nine QTL found across the six chromosomes, of these, *qDMR3.1*, *qDMR5.1*, and *qDMR6.1* being the major QTL. We then mined the putative candidate genes for three major QTL. Together, the results disclose the genetic basis of SDM resistance and setting the groundwork for the map-based cloning of the gene (s) underlying the major QTL detected in this study and are helpful for marker-assisted selection in maize breeding programs.

## Data Availability

The raw data supporting the conclusions of this article will be made available by the authors, without undue reservation.
